# Mine 4.0-mineCareerDB: A high-resolution image dataset for mining career segmentation and object detection

**DOI:** 10.1016/j.dib.2024.110976

**Published:** 2024-10-01

**Authors:** Nasreddine Haqiq, Mounia Zaim, Mohamed Sbihi, Khalid El Amraoui, Mustapha El Alaoui, Lhoussaine Masmoudi, Hamza Echarrafi

**Affiliations:** a Laboratory of Systems Analysis, Information Processing and Industrial Management (LASTIMI), High School of Technology of Salé, Mohammed V University in Rabat, Rabat, Morocco; b LCS Laboratory, Physics Department Faculty of Science, Mohammed V University in Rabat, Rabat, Morocco; c SGMG Company, Co-Founder, Kenitra, Morocco

**Keywords:** Mining industry, Image classification, Drone imagery, Image dataset, Computer vision, Deep learning

## Abstract

The article presents Mine 4.0-MineCareerDB, a publicly available dataset of high-resolution image captured by a DJI Phantom 4 RTK drone specifically designed for analyzing mining careers. The dataset comprises a collection of 373 images depicting various mining operations and activities. Each image is georeferenced and offers a detailed view of mining activities, including the use of various equipment, infrastructure, and overall mining environment. This dataset has the potential to be a valuable resource for computer vision applications in the mining industry such as developing algorithms for identifying mining equipment, training deep learning models for safety analysis and optimization, and research on automation in mining operations. By making Mine4.0-MineCareerDB publicly available, we aim to stimulate further advancements in computer vision research and its applications in the mining sector. The dataset is available at: https://data.mendeley.com/datasets/c5s76mj4bm/5

Specifications Table

**Table:** 

Subject	Computer Vision Applications (CV).
Specific subject area	Mining 4.0, Deep Learning, Object Detection.
Type of data	RGB Images, LASer Format (LAS), Tag Image File Format (TIFF)
Data collection	The data were collected using DJI Phantom 4 Unmanned Aerial Vehicle (UAV) equipped with its standard high-resolution FC6310 camera of 5472 × 3648 pixels. Flight paths for the UAV were meticulously planned to ensure coverage of the Benslimane mine site including active pits, haul road and processing facilities. During the data collection period, georeferenced images were captured over a flight time of approximately 30 min using the UAV's GPS system. This ensures each image can be accurately positioned within the spatial context of the mine site. The data collection area has −7.02° of latitude, 33.64° of longitude and a flight altitude of 398 m.
Data source location	City / Town / Region: Benslimane Career site Country: **Morocco** GPS coordinates of the mining career: 33.64142489702426, −7.023317383836225 or 33°38′29.1″N 7°01′23.9″W
Data accessibility	Repository name: MineCareerDB Data identification number: doi: 10.17632/c5s76mj4bm.5 Direct URL to data: https://data.mendeley.com/datasets/c5s76mj4bm/5 Instructions for accessing these data: Click on the link in part of Direct URL to data
Related research article	N. Haqiq, M. Zaim, M. Sbihi, M. El Alaoui, L. Masmoudi, H. Echarrafi, “An improved mining image segmentation with K-Means and morphology using drone dataset “. International Journal of Electrical and Computer Engineering (IJECE), vol. 14, no. 3, pp. 2655–2675, June 2024, doi: 10.11591/ijece.v14i3.pp2655–2675 [1].

## 1 Value of the Data

The Mine 4.0-MineCareerDB dataset offers a valuable resource for research in computer vision and mining engineering. Here are some key aspects that highlight its significance:

• *Unique focus on mining operations:* The dataset specifically focuses on high-resolution drone imagery of mining activities. This targeted approach provides researchers with a valuable resource for developing and testing computer vision algorithms.
• *Applications in Object Detection and Classification*: The detailed visual data allows researchers to train algorithms for task like automatic identification and classification of mining equipment, infrastructure elements and activities such as drilling and hauling.
• *AI applications on Object Detection:* The rich information captured in the images has the potential to be used for various purposes. Researchers could explore areas like activity recognition with analyzing traffic patterns of mining vehicles, environmental monitoring with dust control and even 3D reconstruction of mine sites for planning and analysis.
• *Algorithm Development:* The dataset conserved as an evaluation of the performance of computer vision algorithms designed for mining applications. Researchers can compare their algorithms accuracy and effectiveness on this dataset.

## 2 Background

The dataset Mine 4.0-MineCareerDB was created in response to the growing demand for high-quality visual data in computer vision for mining applications. Advancements in computer vision offer exciting possibilities for growing safety and efficiency in various industries and the necessity for robust algorithms like real-time world mining environments. Public datasets focused on mining operations are limited and MineCareerDB aims to fill this gap by providing high quality resolution drone images that capture mining activities*.*

## 3 Data Description

The paper presents a dataset of high-resolution captured by drone DJI Phantom 4 at the Benslimane site in Morocco. There are 373 individual RGB image files of 5472 × 3648 pixels. To facilitate object identification and analysis, 203 of these images have been annotated using platforms as makesense.ai and apeer. These annotations categorize objects within the images into four distinct classes: trucks, mining equipment or materials, cars and persons.

In Addition to the annotated imagery, the dataset includes two geospatial files generated using Agisoft Metashape software. These files are:

- **LAS File:** This file represents a single 3D point cloud generated from the entire set of 373 high-resolution images captured by DJI phantom4 drone. The Point cloud provides a detailed representation of the 3D structure of the Benslimane mine site. Although the dataset includes 373 images, the LAS file consolidates information from all of them into a single point cloud, rather than having a separate LAS file for each image.
- **DEM File:** This file is a Digital Elevation Model derived from point cloud data. It provides a topographical representation of the terrain.

The remaining 170 images not annotated, contribute significantly to the dataset by providing essential data for constructing the 3D model of the mine, including the generation of the point cloud and the digital elevation model (DEM). While these images do not contain objects of interest for annotation, they are essential for accurately modeling the terrain and structure of the mine. The LAS/DEM files in the MineCareerDB dataset serve as essential tools for spatial analysis, complementing the annotated images. The LAS file provides a 3D point cloud data that is used for precision mapping of annotated objects within the mine structure, facilitating tasks such as measuring distances between objects, assessing the spatial distribution of annotated features, and understanding the overall structure of the mine site. The DEM file provides a topological context, allowing to identify slopes, elevation, and depressions.

The relationship between the LAS/DEM files and the annotations is straightforward for spatial analysis within the MineCareerDB Dataset. The annotations categorize and pinpoint specific objects within the images such as trucks, mining equipment, cars, and persons. By combining the annotated images within LAS and DEM, we can accurately map the precise locations of each object within the 3D structure of the mine terrain, analyze their spatial distribution, and examine how the topography may affect their placement or movement.

**Fig. 1:**
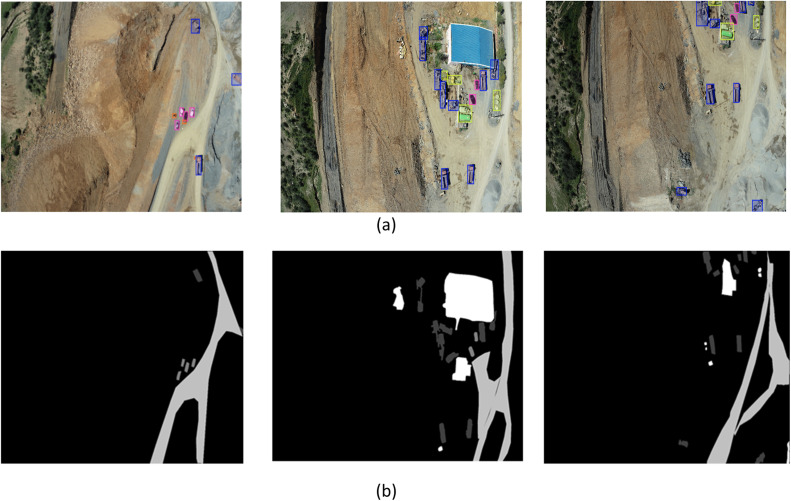
Collection of sample images with some examples of annotated images. (a) Example of annotated images by make-sens.ai. (b) Example of annotated images by apeer.com.

To provide a clearer understanding of the dataset's composition, Fig. 1 presents a collection of sample images while Table 1 summarizes the key components of the dataset. It details the number of high-resolution images captured, the consistent naming format used for each file and the image resolution. Table 2 focuses on the technical specifications of the camera used for data collection. This table provides details about the camera model and sensor type.

**Table 1:** The key components of the dataset.

Folder	Filename	Feature	Description
MineCareerDB	DJI_XXXX.JPG	Image files	373 high-resolution images
Other Data	DEM_Benslimane.tif	TIF file	Digital Elevation Model
	Benslimane_point_cloud.las	LAS file	3D point cloud file
Annotation/Images	XXX.jpg	Image files	RGB images (000 – 202)
Annotation/label	XXX.xml	XML files	VOC format (000 – 202)
	XXX.xml	XML files	YOLO format (000 – 202)
Annotation/Apeer	XXX.tiff	TIFF files	Annotations (000- 202)

**Table 2:** Technical characteristics of FC6310 Camera.

FC6310 Camera						
Sensor	Effective pixel	Lens	Image resolution	ISO range	Max bitrate	Image formats
1 in. CMOS	20 Megapixels	FOV 84°,8.8 mm	5472 × 3648	100–3200 (Auto)	100 Mbps	JPEG, DNG (RAW)

The annotated images serve as a valuable resource for training algorithms to automatically identify and classify different features with mining site. Table 3 summarizes the key statistics of different datasets in the mining industry. Seg refers to segmentation and Clas refers to classification.

**Table 3:** datasets statistics for mining industry images.

Dataset	Images	Size	Labels	Tasks	Image size (Pixels)	Channels	Resolution (GSD)
Bridgeport Moreland Construction [2]	385	7.78 GB	N/A	Seg & Clas	9504 × 6336	RGB	2.3 cm/px
Serbia Kolubara Mine [3]	362	6.23 GB	N/A	Seg	7952 × 5304	RGB	3.2 cm/px
UAV-PDD2023 [4]	535	2.1 GB	1070	Seg & Clas	5472 × 3648	RGB	2.5 cm/px
**MineCareerDB (our)**	**373**	**2.99 GB**	**203**	**Seg & Clas**	**5472 × 3648**	**RGB**	**2.5cm/px**

The MineCareerDB dataset stands out from other datasets in several ways. Unlike the Bridgeport Moreland Construction and Serbia Kolubara Mine datasets, which do not include labeled annotations, our dataset provides 203 annotated images that classify objects into specific categories. This annotation makes MineCareerDB particularly valuable for both segmentation and classification tasks, enabling more advanced and precise machine learning applications.

Additionally, MineCareerDB maintains a high resolution (2.5 cm/px), similar to UAV-PDD2023, but with a specific focus on mining site analysis. While UAV-PDD2023 has a larger number of images and labels, MineCareerDB offers a specialized dataset tailored for the mining industry, including comprehensive geospatial data such as LAS and DEM files. These files provide 3D spatial context and terrain information, facilitating the analysis of the annotated objects within the physical environment, a feature not emphasized in the other datasets.

Moreover, MineCareerDB integrates detailed object-level annotations and broader site features through platforms like MakeSenseAI and Apeer, enhancing its utility in environmental impact assessments and mining site management. This detail makes the dataset a unique resource for researchers and practitioners in the mining industry.

## 4 Experimental Design, Materials and Methods

1. *Flight Planification and data acquisition*:

The flight planning began with identifying the geographical area of interest, which was a mining site with diverse terrain and features. The area was selected based on the presence of distinct geological structures, which are critical for testing effectiveness and segmentation methods. Different parameters should be taken into consideration:

- *Weather:* weather conditions were monitored using local weather stations and forecasts to ensure optimal flying conditions. Flights were scheduled during periods of clear weather with minimal wind speed to avoid disturbances that could affect the drone's stability or the quality of the image.
- *Georeferencing:* Images were referenced using Ground Control Points (GCPs) placed throughout the mining site. These GCPs were surveyed with high-precision GPS equipment, ensuring accurate spatial positioning of the images.
- *Safety protocols:* To ensure safety during the flights, we verified that the drone's battery was charged fully before the mission started. The environment was thoroughly studied to ensure it was suitable for drone operations, avoiding areas with potential hazards such as strong winds that could damage the drone. A backup drone was always kept ready to deploy in case of any technical issues were encountered, ensuring

For our data acquisition, we employed a DJI Phantom 4 Pro drone, equipped with a 20 megapixels camera featuring a 1-inch CMOS sensor achieving a Ground Sample Distance (GSD) of approximately 2.5 cm/pixel. Flight parameters were meticulously set with an altitude of 398 m above ground level, speed of 6 m s^-1^, and image overlaps of 80 % frontal and 70 % side to ensure comprehensive coverage. High-precision GPS equipment was used for surveying GCP's to guarantee accurate georeferencing of the images. Table 4 presents the parameters of mission acquisition.

2. *Data annotation*:

**Table 4:** Parameters of the acquisition mission.

Flight altitude	398 m
**Estimated resolution**	2.5 cm/px
**Surface Area Covered**	2 km²
**Flight time**	30 min
**Number of batteries**	2
**Weather conditions**	clear
**Spectral bands**	RGB
**Speed**	6 m s^-1^

The collected mining site data was annotated by a team of geological experts to ensure the accuracy and relevance of the annotations. This process was crucial for creating a high-quality dataset suitable for various machine learning applications in the mining industry. Two online platforms, Apeer and makesenseai, were used for the annotation tasks, each serving distinct purposes to maximize utility of dataset:

- *MakeSenseai Platform* [5]**:** using MakeSense, the images were annotated to classify different regions based on geological features. The annotation included four classes: trucks, materials, cars and persons. Annotators used polygon outlines to precisely delineate the boundaries of these regions, ensuring a detailed and accurate annotation.
- *Apeer Platform* [6]**:** Apeer was employed for annotating structures within the mining site, categorizing them into four classes: road, trucks, cars, and materials. Similar to the MakeSense annotations, polygon outlines were used to capture the exact shapes and sizes of the structures. This classification is particularly useful for environmental impact assessments and for developing models to predict materials and structure types in and around the mining area.

The use of both MakeSenseAI and Apeer platforms for annotation in the MineCareerDB dataset was intentional, leveraging the distinct strengths of each tool. MakeSenseAI is particularly well-suited for annotating specific objects within images such as trucks, materials, cars, and people, providing a user-friendly interface for precise polygon-based annotations. It's ideal for clearly delineating well-defined objects within the mining site. On the other hand, Apeer is excellent at handling more complex and structured annotations such as roads and larger site features, focusing on categorizing extensive areas and structures. Apeer's advanced capabilities in managing hierarchical and multi-class annotations make it indispensable for capturing the broader context and environmental structures within the mining site.

Using both platforms is necessary to ensure a comprehensive and accurate annotation across the dataset. MakeSenseAI is ideal for detail object-level annotations, while apeer allows for the annotation of larger and more complex structures. This combination ensures that the dataset includes both fine-grained details and broader contextual information, which is crucial for developing robust machine learning models that can accurately identify and classify various features within the mining environment.

3. *Point cloud and Digital Elevation Model production*:

The production of the point cloud was carried out using Agisoft Metashape [7], a photogrammetry software specialized in creating 3D models from aerial imagery. The process began by importing the high-resolution images acquired during drone flights into metashape. The images were aligned through a feature matching that identifies and matches common points across overlapping images. This step is important for reconstructing the 3D geometry of the scene. Once the image alignment was completed, a sparse point cloud was generated which represents the 3D initial structure [8]. This sparse point cloud is then densified to create a dense point cloud. The densification process involved calculating the depth information for each pixel, resulting in a highly detailed and accurate 3D representation of the mining site. The dense point cloud ensures to remove any noise and to provide a final model quality and precision.

Following the generation of dense point cloud. The next step was to produce the Digital Elevation Model (DEM). The DEM represents the bare-earth surface, excluding objects like trees and buildings for better terrain topography understanding [9]. Once the ground points were isolated, they were used to interpolate a continuous surface, this process involved fitting a surface to the ground points, resulting in a raster grid that represents the terrain's elevation.

**Fig. 2:**
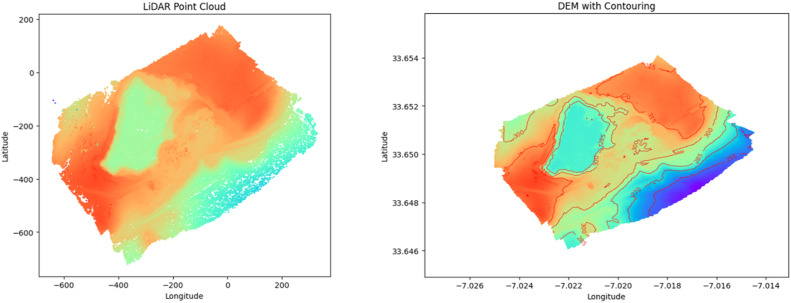
Illustrates the detailed point cloud and the resulting of Digital Elevation Model.

The combination of the point cloud and DEM offers a comprehensive 3D and 2D representation of the site. Fig. 2 illustrates the detailed point cloud and the resulting DEM produced using Agisoft Metashape.

## Limitations

None.

## Ethics Statement

The authors have read and follow the ethical requirements for publication in Data in Brief. This work does not involve human subjects, animal experiments, or data collected from social media platforms.

## Credit Author Statement

**Nasreddine Haqiq:** Conceptualization, Methodology, Software, Data curation, Writing and Validation. **Mounia Zaim**: Supervision, Project administration, Validation, Funding acquisition and Review & Editing. **Mohamed Sbihi**: Validation, Resources, Supervision and Review & Editing. **Khalid El Amraoui:** Software, Investigation, Writing, Data curation and Supervision**. Mustapha El Alaoui:** Supervision, Data curation, Validation, Writing and Review & Editing. **Lhoussaine Masmoudi:** Conceptualization, Supervision, Review & Editing. **Hamza Echarrafi**: Resources, Conceptualization and Investigation.

## Data Availability

Mine4.0 - MineCareerDB : A mining career dataset (Original data) (Mendeley Data).
